# Circulating cell-free DNA profiling reveals ancestry-dependent genetic variation in metastatic prostate cancer

**DOI:** 10.1186/s43556-026-00405-8

**Published:** 2026-02-12

**Authors:** Samaneh Maleknia, Rebecca Hassoun, Nabil Adra, Reza Shahbazi

**Affiliations:** 1 Division of Hematology/Oncology, Department of Medicine, Indiana University School of Medicine, Indianapolis, IN USA; 2 Tumor Microenvironment & Metastasis, Indiana University Melvin and Bren Simon Comprehensive Cancer Center, Indianapolis, IN USA; 3 Brown Center for Immunotherapy, Indiana University School of Medicine, Indianapolis, IN USA

**Keywords:** Prostate cancer, Circulating cell-free DNA, Genetic variations, Somatic mutations, cfDNA profiling, MCRPC

## Abstract

African American men experience markedly higher prostate cancer incidence and mortality yet remain underrepresented in genomic studies aimed at identifying ancestry-associated molecular drivers of disease progression. Circulating cell-free DNA (cfDNA) provides a minimally invasive means of capturing tumor-derived genomic alterations and enables broad characterization of molecular heterogeneity. In this study, we performed comprehensive plasma cfDNA profiling in 22 African American and 66 Caucasian men with metastatic castration-resistant prostate cancer (mCRPC) to define ancestry-related differences in mutation landscapes. Across samples, we identified 281 variants within 88 genes, with African American patients exhibiting a significantly higher burden of differentially frequent mutations. Integration of Gene Ontology analysis, KEGG pathway mapping, and functional enrichment revealed ancestry-dependent perturbations in pathways central to prostate cancer biology, including PI3K–AKT, MAPK, androgen signaling, DNA repair, and cell-cycle regulation. A panel of 25 genes emerged as key contributors to divergent disease mechanisms across racial groups. Several variants enriched in African American patients involved genes regulating genomic stability and treatment resistance, offering potential insight into observed clinical outcome disparities. These findings provide a comprehensive, ancestry-informed cfDNA mutational landscape in mCRPC and highlight biologically meaningful differences that may support the development of non-invasive biomarkers, improved risk stratification, and ancestry-tailored precision oncology strategies.

**Supplementary Information:**

The online version contains supplementary material available at 10.1186/s43556-026-00405-8.

## Introduction

Prostate cancer is the most common cancer diagnosis in U.S. men, accounting for ~ 30% of male cancers in 2025, and remains the second leading cause of cancer death in men. U.S. incidence trends have reversed from a decline (2007–2014) to an increase (2014–2021), with the rise driven largely by regional and distant-stage disease, highlighting a growing burden of advanced presentation. Globally, prostate cancer caused an estimated ~ 397,430 deaths in 2022, emphasizing the need for improved treatments for lethal metastatic disease [1]. African American men (AAM) men experience ~ 67% higher incidence and approximately two-fold higher mortality than Caucasian men (CM) in the United States, reflecting persistent disparities driven by multilevel factors including structural inequities, differential access to high-quality care, and biologic contributors [1]. Recent epidemiological and genomic studies have highlighted that ancestry-associated biological differences, including distinct germline predispositions and somatic alterations, may contribute significantly to these outcome gaps. Yet, racial and ethnic minority groups remain systematically underrepresented in genomic sequencing studies, limiting our understanding of ancestry-related molecular drivers of PC progression and treatment response [2, 3].

Parallel to these disparities, cfDNA has emerged as a minimally invasive and scalable modality for characterizing tumor genomics, monitoring disease progression, and identifying mechanisms of treatment resistance [4, 5]. Plasma cfDNA profiling captures real-time tumor heterogeneity and has shown utility in metastatic PC for detecting actionable variants, monitoring clonal evolution, and predicting therapeutic response [6, 7]. Despite this potential, its genome-wide application to investigate ancestry-specific genomic variation in advanced PC remains insufficiently explored. Most prior studies have relied on restricted, predefined targeted panels that may miss rare, population-specific variants, particularly in underrepresented groups such as AAM.

Given the urgent need for equitable, ancestry-informed cancer genomics, recent investigations have emphasized the importance of including diverse populations to uncover molecular mechanisms underlying racial differences in PC incidence and outcomes [8–10]. To address this unmet need, we performed a targeted, broad-scope cfDNA sequencing approach to capture the full spectrum of potential genetic alterations in mCRPC. This approach enables the detection of both common prostate cancer–related alterations and rare population-specific variants, offering a more comprehensive understanding of molecular disparities between AAM and CM patients.

In this study, we hypothesized that AAM and CM patients with mCRPC harbor distinct germline- and somatic-derived cfDNA mutation patterns that contribute to differences in disease biology and may inform ancestry-tailored diagnostics. To test this hypothesis, we systematically profiled plasma cfDNA from AAM and CM patients and integrated these findings with two external validated datasets: (i) COSMIC, to determine whether identified mutations represent known somatic cancer alterations, and (ii) gnomAD, to evaluate ancestry-related allele frequencies. We then mapped these variants onto prostate cancer–related signaling pathways and conducted functional enrichment analyses to identify divergent biological processes. The study’s overarching objective is to define population-specific genomic signatures and evaluate their potential as non-invasive diagnostic or prognostic biomarkers for AAM and CM patients with mCRPC. Our findings reveal ancestry-linked genetic differences involving key pathways such as PI3K–AKT, MAPK, P53, and androgen signaling, underscoring the clinical importance of incorporating ancestry context into precision oncology efforts for prostate cancer.

## Results

### Overview of cfDNA mutation profiling and cross-racial comparative analysis

**Fig. 1:**
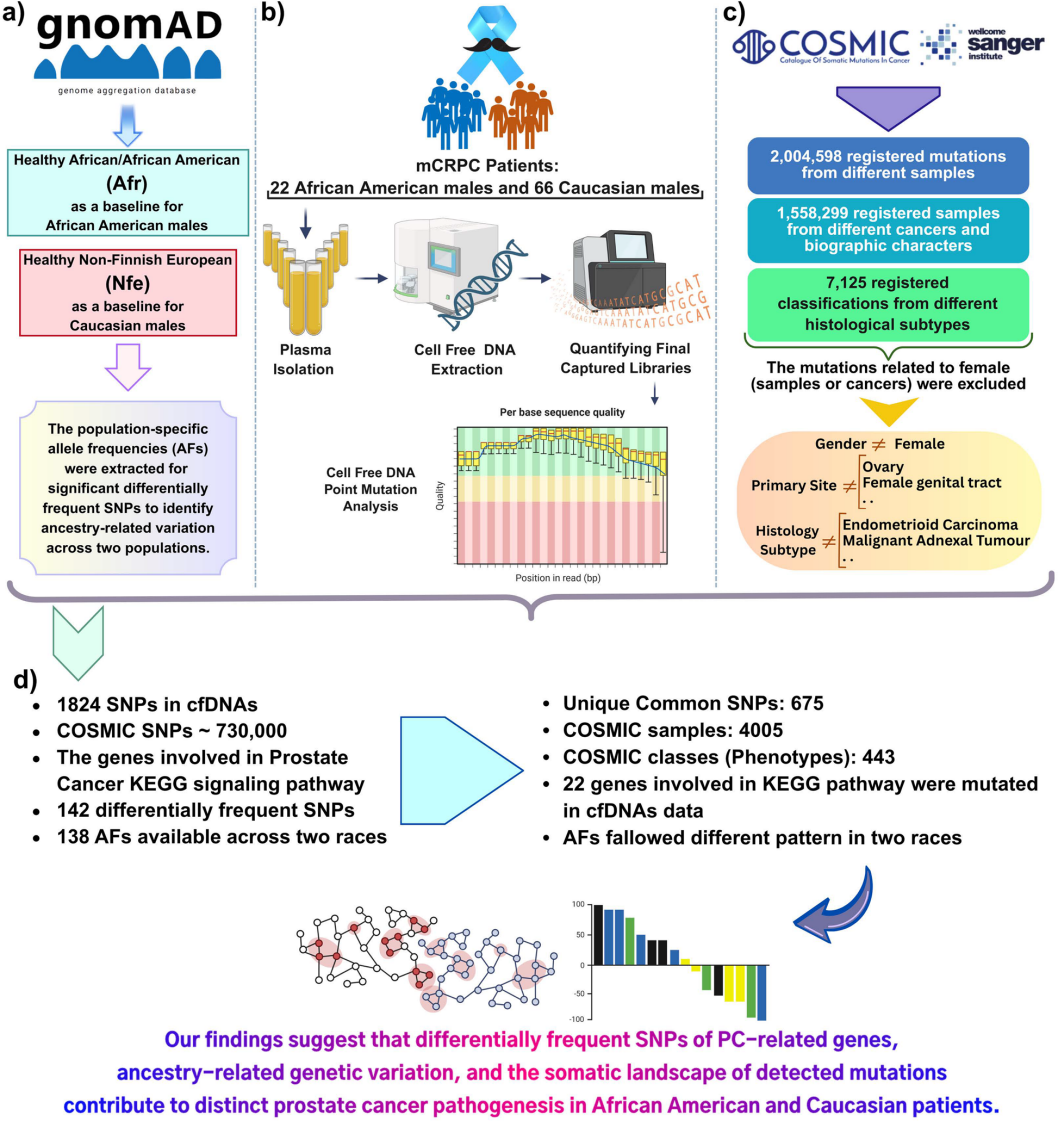
Overview of the analytical workflow. **a** Population-specific allele frequencies were retrieved from the gnomAD database to assess ancestry-associated variation among the detected variants. **b** Cell-free DNA was isolated from plasma samples and used to identify and characterize genomic alterations across individuals. **c** Variant-level information was further evaluated using the Catalogue of Somatic Mutations in Cancer (COSMIC) to determine whether the detected mutations had been previously reported in other tumor types. **d** Comparative integration of cfDNA-derived variants with COSMIC annotations identified multiple shared SNPs, including those classified as somatic mutations

To delineate the variations in mCRPC pathogenesis pathways between two racial groups, cfDNAs were isolated from plasma samples and subsequently used to construct mutation libraries. Statistical and bioinformatics methodologies were then applied to analyze the identified genetic variances across the samples (Fig. 1b). Population-specific allele frequencies (AFs) were obtained from the gnomAD database to evaluate ancestry-associated variation among the significantly differentially frequent single nucleotide polymorphisms (SNPs). The gnomAD African/African American (Afr) and Non-Finnish European (Nfe) populations were used as baselines for the AAM and CM patient cohorts, respectively (Fig. 1a).

To further validate the findings, corresponding data from the Catalogue of Somatic Mutations in Cancer (COSMIC) database were used to ascertain which of the detected mutations had been previously reported in other tumor types. After applying specific inclusion and exclusion criteria, approximately 730,000 unique SNPs extracted from COSMIC were utilized for downstream analysis (Fig. 1c). Comparison of cfDNA-derived variants with the selected COSMIC SNP set identified 675 shared SNPs, of which 394 were classified as somatic mutations. (Fig. 1d).

This study illustrates potential differences in prostate cancer pathogenesis across AAM and CM patients. Although the sample size constrains the strength of statistical inference, the patterns observed here uncover biologically compelling signals that advance current understanding and establish a strong framework for future mechanistic and large-scale validation studies.

### Sample characteristics and demographics for cross-racial analysis in prostate cancer metastasis

The results presented are derived from cross-sectional data obtained from samples that fulfilled the inclusion and exclusion criteria. One sample was initially omitted due to inadequate sequencing quality, while two samples were excluded due to absent racial information. This culminated in a final curated dataset of 88 plasma samples, consisting of 66 from CM and 22 from AAM. The average age ± standard deviation (SD) for CM and AAM samples was 62.3 ± 7.9 years and 59.45 ± 8.4 years, respectively. The samples in both groups were age-matched, and statistical analysis via a t-test indicated no significant age difference between the groups (*P*-value = 0.1738).

Most patients had documented histories of metastasis, including locations and timelines. The predominant location of metastasis in both cohorts was the bone. This indicates that subsequent to the development of prostate cancer, bone tissue has emerged as the primary location for the commencement of metastasis in both racial groups (Table 1. Part 1).

**Table 1:** The frequency of patients in any metastatic site and time category

Part 1: Metastatic site. LN: lymph node			Part 2: Metastatic Time		
Metastasis Sites	Race	Frequency	Metastasis Time	Race	Frequency
		(Percentage)			(Percentage)
Bone	Caucasian	27(40.9)	At Diagnosis	Caucasian	24(36.4)
	African American	6(27.3)		African American	13(59.1)
Regional LN, Distant LN, Bone	Caucasian	9(13.6)	At Relapse	Caucasian	38(57.6)
	African American	6(27.3)		African American	8(36.4)
Regional LN, Distant LN	Caucasian	9(13.6)	Not Reported	Caucasian	4(6)
	African American	1(4.5)		African American	1(4.5)
Regional LN, Bone	Caucasian	7(10.6)			
	African American	4(18.2)			
Not Reported	Caucasian	6(9.1)			
	African American	3(13.6)			
Liver, Bone	Caucasian	3(4.5)			
	African American	0(0)			
Regional LN	Caucasian	2(3)			
	African American	1(4.5)			
Bone, Brain	Caucasian	1(1.5)			
	African American	0(0)			
Distant LN	Caucasian	1(1.5)			
	African American	0(0)			
Lungs, Bone	Caucasian	1(1.5)			
	African American	0(0)			
Regional LN, Liver, Bone	Caucasian	0(0)			
	African American	1(4.5)			

We recorded the timing categories of metastasis, encompassing diagnosis or relapse, for the patients (Table 1. Part 2). A higher proportion of AAM exhibited metastasis at diagnosis, while CM predominantly reported metastasis at relapse. Nonetheless, this result did not achieve statistical significance (*P*-value = 0.061457). No significant difference exists between the two patient groups regarding demographic and clinical data.

### Analysis of genetic variants by functional consequences, significance, and chromosomal distribution

To gain insights into cfDNA profile changes among different races of PC patients, we analyzed FASTQ files from 88 plasma samples. The final VCF data, used for identifying genetic variants in each sample, was obtained through *Partek® Flow®* software. A total of 1824 variants within 150 genes or loci (like LOC105379418) met all criteria and demonstrated acceptable quality for inclusion in the analysis (Table S1). Among these variants, 77 genes and loci with notable mutations showed no significant difference in proportions between the two ethnic groups that among them mutations related to p53 were detected. In contrast, 142 variants across 76 genes and loci exhibited significantly different mutation frequencies between AAM and CM (Table S1).

A total of 1,376 variants did not receive an amino-acid change (AA_CHANGE) annotation. This outcome is expected given the biological context of cfDNA variant profiles. The majority of detected variants are noncoding, including intronic variants, UTR variants (U3, U5), intergenic variants, synonymous substitutions that do not alter protein sequence, and other regulatory or intergenic alterations. Such variants inherently lack a protein-level consequence, and therefore AA_CHANGE is appropriately recorded as NA. Additionally, some variants could not be annotated due to limitations in available transcript models and incomplete external reference annotations. ClinVar-derived annotations (CLNHGVS) are also limited, and many variants lack ClinVar entries; consequently, amino-acid change information could not be obtained even after fallback parsing.

**Fig. 2:**
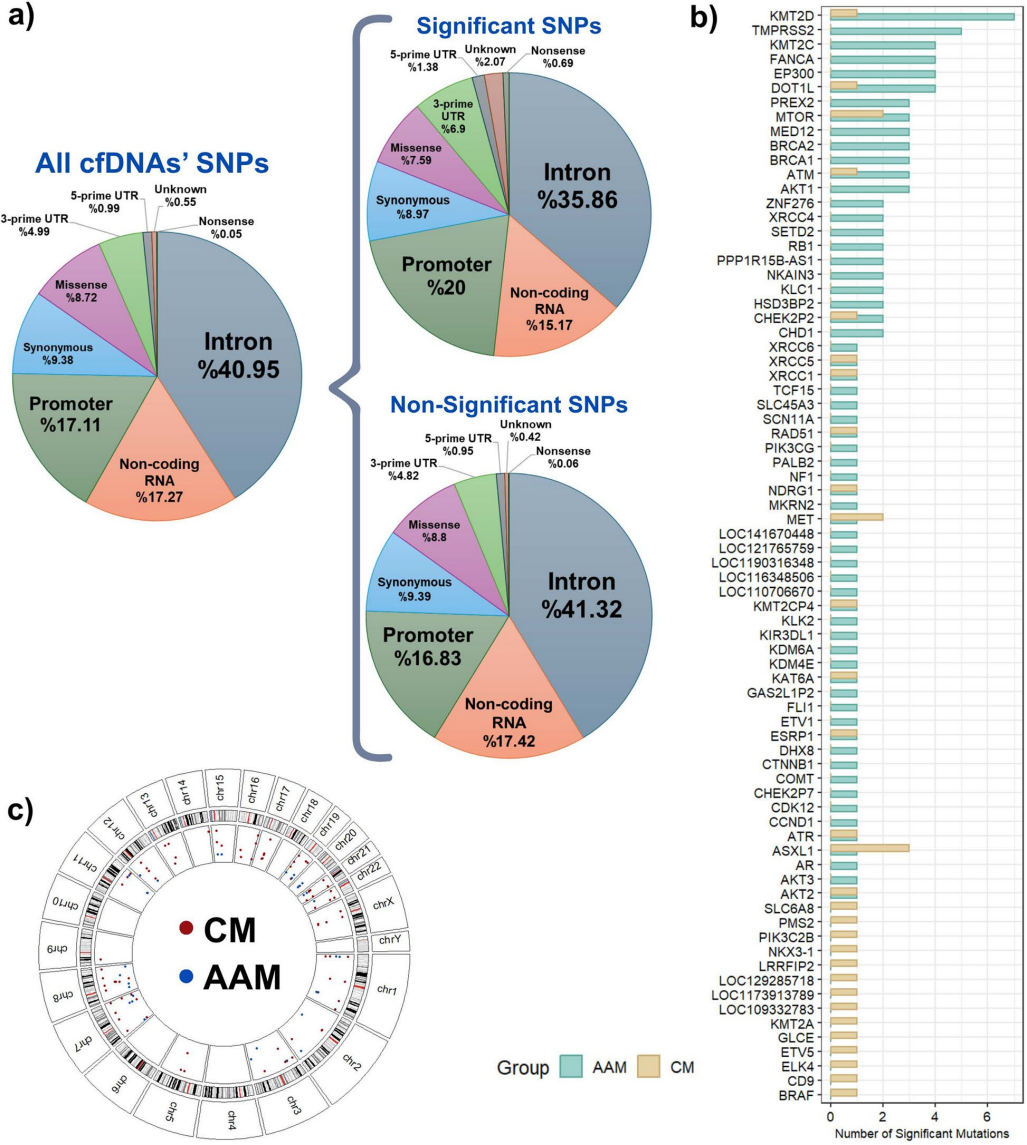
Comparative analysis of variant characteristics, functional consequences, and chromosomal distributions between racial groups. **a** Proportions of functional consequences among all, significant, and non-significant variants. **b** Distribution of significant mutations across genes predominantly altered in African American (AAM)-dominant (Green) and Caucasian (CM)-dominant (Beige) groups. **c** Chromosomal distribution of significant variants

The analysis of functional consequence proportions across all variants, significant variants, and non-significant variants revealed no significant differences in these ratios (Fig. 2a). The majority of variants were located in intronic regions, non-coding transcripts (such as non-coding RNAs), and promoter regions. Further analysis of significant variant proportions indicated no significant differences between the two racial groups (*P* = 0.61). Consequently, the functional consequences of the variants are consistent across different racial groups.

Notably, the genes *KMT2D, FANCA, DOT1L, MTOR, TMPRSS2, ASXL1, ATM, EP300,* and *KMT2C* showed the highest number of significant differences between the two groups with 9 to 4 significant variations (Fig. 2b). A significant difference was observed in the distribution of variants between the two ethnic groups, with a higher burden of significant variants detected in the AAM cohort. A total of 108 SNPs displayed significantly higher frequencies among AAM patients, in contrast to the 34 mutations found to be predominant in CM patients (Table S1, Significant Variants Sheet).

An analysis of the full dataset revealed a non-uniform distribution of detected variations across the human genome. Chromosome 7 exhibited the highest density of variations, with a total of 193 SNPs identified. In contrast, no variations were identified on chromosome 18 within our dataset. Only four distinct SNPs were detected on the Y chromosome, all associated with the *CHEK2P1* gene locus.

When focusing specifically on SNPs that were significantly predominant between AAM and CM populations, we observed distinct chromosomal patterns as well (Fig. 2c). Chromosomes 1, 7, and 21 each contained 13 significant variants, while chromosome 8 harbored 12 significant variants. These predominant variants localized to a total of 30 distinct genes and genomic loci, including key PC-related genes such as TMPRSS2, MTOR, MET, NKX3-1, AKT3, KMT2C, BRAF, PIK3C2B, PIK3CG, and ETV1.

Conversely, several autosomal chromosomes, namely chromosomes 4, 6, 10, and 18, lacked any significant population-specific variants. Furthermore, extensive analysis of the Y chromosome variants revealed no significant differences in allele frequencies between the two racial groups concerning the four identified CHEK2P1 mutations.

### Integration of cfDNA variants with COSMIC reveals somatic mutational landscape of prostate cancer–related genes

To define the somatic landscape of the detected mutations, the COSMIC database was queried. Following the application of the inclusion and exclusion criteria to the COSMIC database, we compared the remaining mutations with our cfDNA dataset.

A total of 675 common SNPs were identified between the two datasets (Table S2). Of these common variants, 394 were confirmed as somatic mutations within the curated COSMIC data, while 281 were categorized as not confirmed somatic (likely germline polymorphisms within the COSMIC dataset).

To specifically identify PC-related variations, we integrated a list of 106 genes involved in the KEGG "Prostate Cancer" signaling pathway into our analysis. Within the common variations between our cfDNA and the COSMIC datasets, 345 mutations related to 18 PC-related genes were identified. This subset included 98 confirmed somatic mutations, and 247 variants not confirmed as somatic. Notably, cfDNA SNPs related to the PC-related genes AKT3, ETV5, KLK3, KRAS, and NKX3-1 were not present in the current filtered COSMIC dataset.

**Fig. 3:**
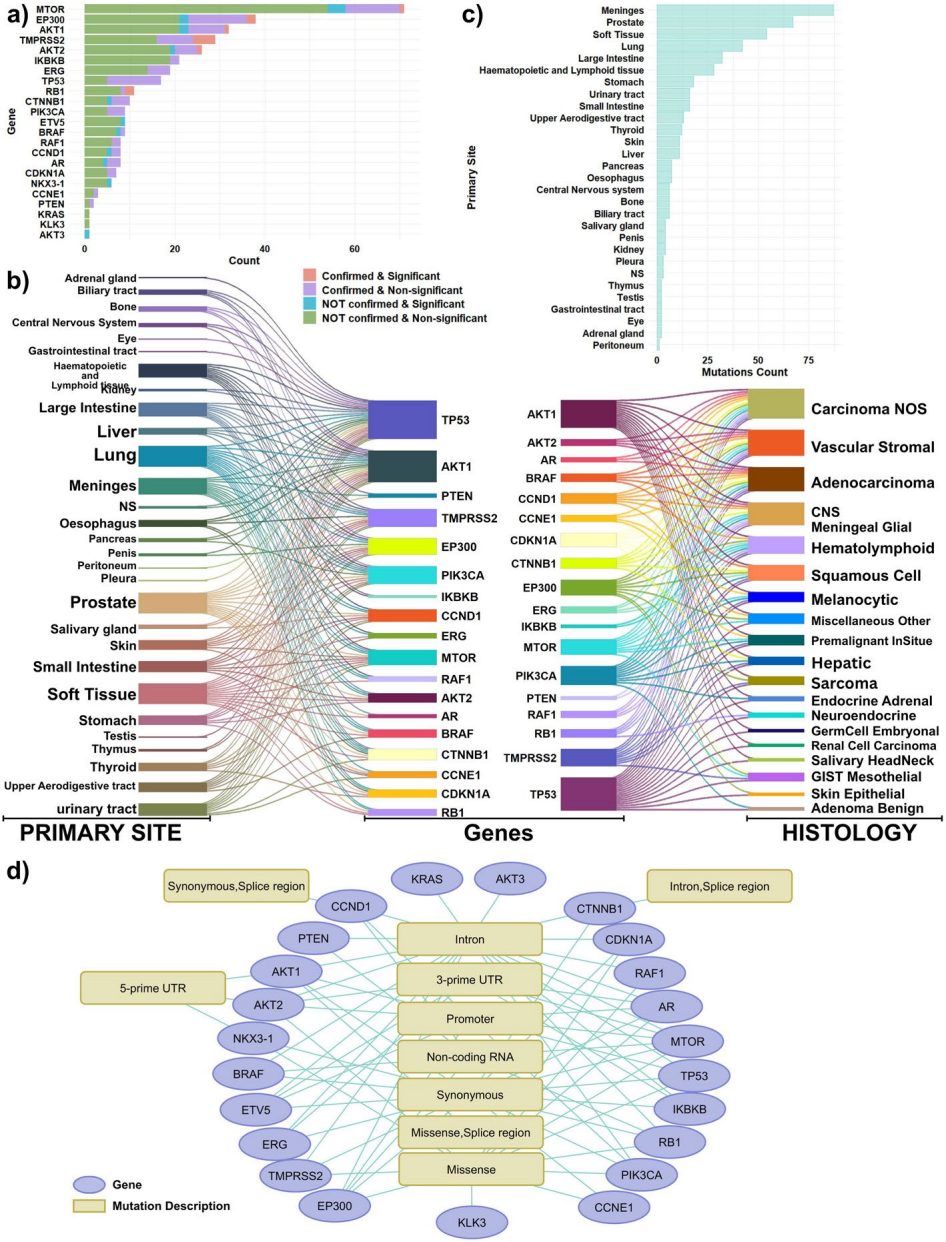
Functional characterization of prostate cancer–related mutations identified in cfDNA. **a** Distribution of significant mutations in genes associated with the KEGG Prostate Cancer signaling pathway, integrating somatic profiles and statistical significance. **b** Distribution of primary tumor sites and histological types for 18 prostate cancer–related genes carrying mutations shared between COSMIC and cfDNA datasets. **c** Distribution of primary tumor sites for significant mutations in the 18 prostate cancer–related genes. **d** Functional consequences of prostate cancer–related genes based on associated SNP annotations

The combined analysis of somatic landscape data and statistical significance for all variations across 23 PC-related genes is summarized in Fig. 3a. The distribution of the primary tumor sites and histologies for the 18 genes with common mutations shared between the COSMIC and cfDNA datasets are exhibited in Fig. 3b. We confirmed that 15 of these 18 genes had at least one corresponding sample extracted from prostate primary site malignancies (Fig. 3c).

Due to the established importance of these 23 PC-related genes in prostate cancer pathogenesis, we further investigated the functional consequences of their associated SNPs. Genes such as KRAS, and AKT3 included only one intronic variation, while KLK3 exclusively showed missense variation. The remaining genes exhibited multiple functional variation types (Fig. 3d).

**Fig. 4:**
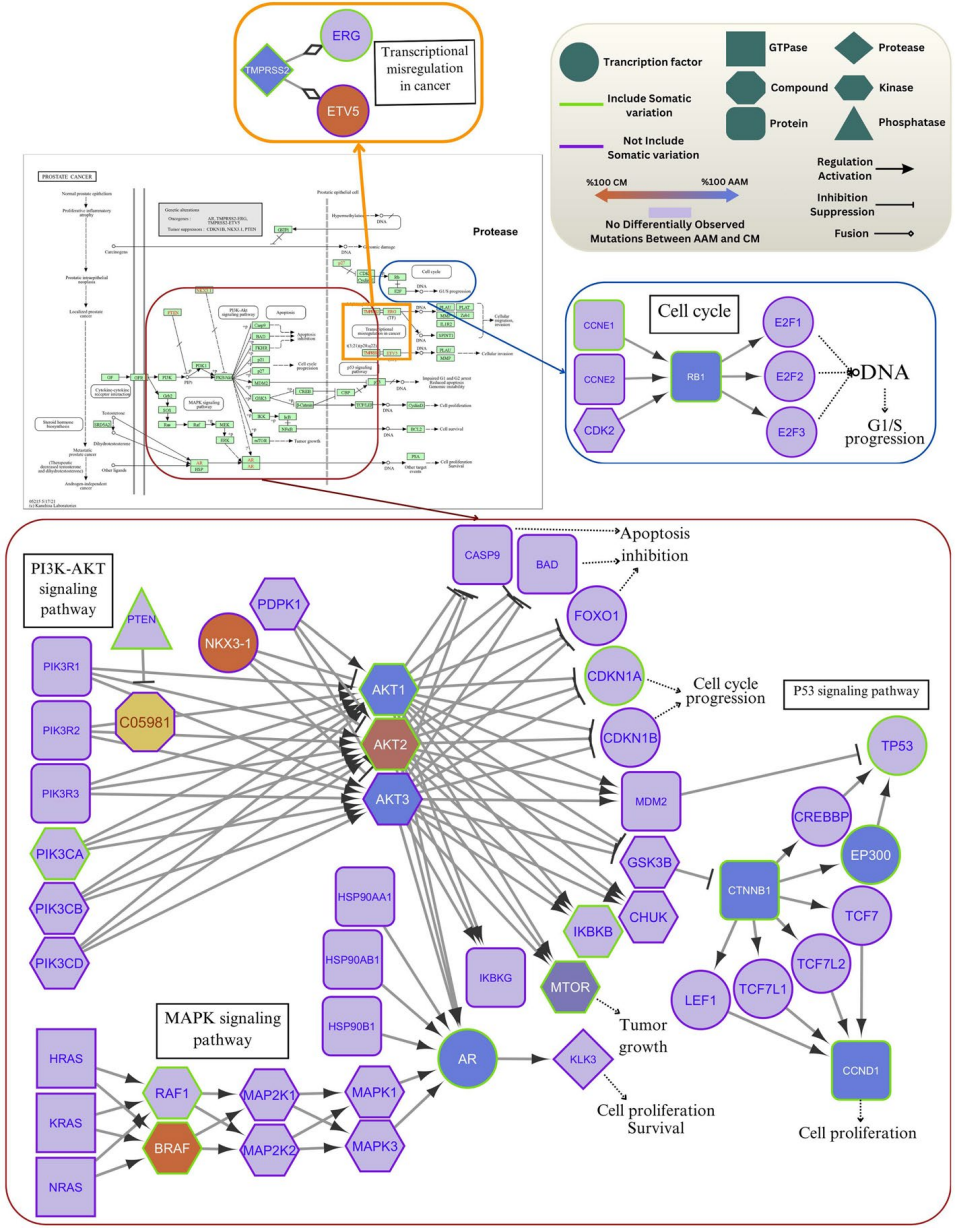
Reconstruction of the KEGG prostate cancer signaling pathway. The pathway was reconstructed by integrating somatic mutation profiles, gene functions, regulatory interactions, and the relative proportions of predominant mutations observed within each racial group

By integrating data regarding somatic variation spectrum, gene function, gene regulation type, and the proportion of predominant mutations within each racial group, we reconstructed the KEGG "Prostate Cancer" signaling pathway. This integrated analysis revealed alterations in three distinct parts of the pathway (Fig. 4). The analysis identified key, interconnected pathways implicated in prostate cancer, including PI3K–AKT, MAPK, p53, cell cycle regulation, and transcriptional dysregulation.

In terms of signaling dynamics, mutations observed in both AAM and CM racial groups primarily induced perturbations in the PI3K-AKT and MAPK signaling pathways. However, the specific dysregulation patterns and somatic mutation profiles varied significantly, involving different driver genes in each cohort. In AAM patients, these pathway perturbations were predominantly driven by mutations in AKT1, AKT3, CTNNB1, EP300, CCND1, MTOR, and AR. Conversely, in CM patients, NKX3-1, AKT2, and BRAF played more prominent roles. These distinct mutations further influenced tumor growth and cell proliferation by disrupting the Cell Cycle and P53 signaling pathways. With the exception of AKT3 and NKX3-1, the genes mentioned above included at least one confirmed somatic mutation within the curated datasets. Within the Cell Cycle pathway component, CCNE and RB1 showed confirmed somatic mutations, with RB1 mutations also being dominant in the AAM cohort.

We next examined mutations in genes implicated in fusion events. KEGG describes TMPRSS2–ERG and TMPRSS2–ETV5 fusions as chromosomal rearrangements that typically arise through deletions or translocations. The TMPRSS2–ERG fusion, a well-established alteration in prostate cancer, places the androgen-responsive TMPRSS2 promoter upstream of ERG, leading to aberrant ERG overexpression and downstream transcriptional dysregulation, consistent with the pathway diagram. In our cfDNA dataset, variants in TMPRSS2 were detected predominantly in the AAM group, whereas variants in ETV5 were more frequent in the CM group. In addition, both TMPRSS2 and ERG harbored somatic mutations within the cfDNA cohort. These findings suggest that mutational burden and pathway-level driver mechanisms differ between racial groups, with AAM patients exhibiting a higher number of potentially impactful mutations associated with prostate cancer progression.

### Cross-cancer genetic convergence in prostate-derived samples emerges from shared mutational landscapes beyond canonical prostate pathways

In this phase of the study, we analyzed variants shared between the COSMIC and cfDNA datasets, even though they are not directly linked to canonical prostate cancer signaling pathways. In total, we identified 497 SNPs mapping to 36 genes.

**Fig. 5:**
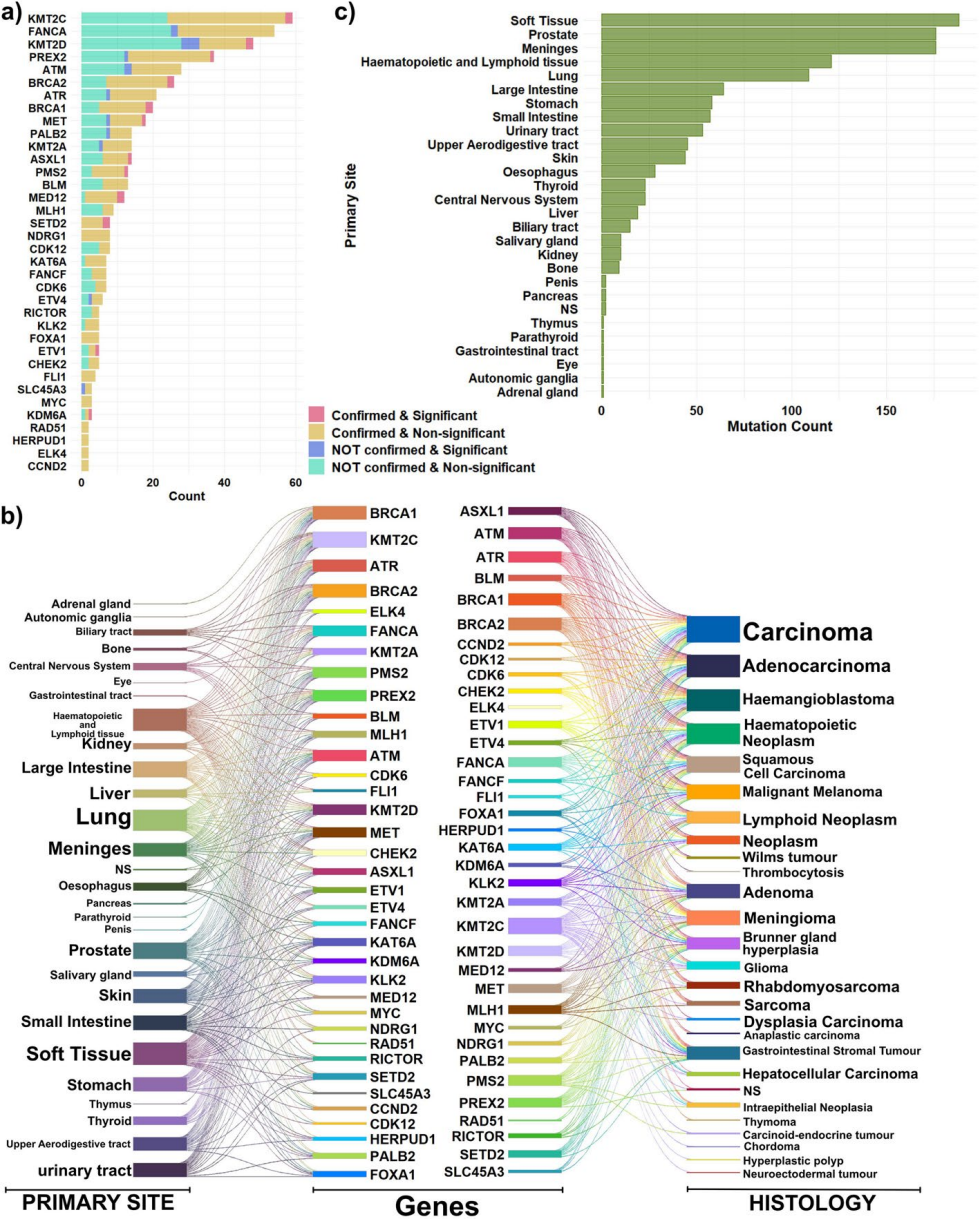
Characterization of mutations shared between COSMIC and cfDNA datasets but not directly implicated in prostate cancer signaling pathways. **a** Distribution of mutations based on the combination of somatic classification and statistical significance. **b** Distribution of primary tumor sites and histological types for 36 genes carrying mutations shared between the COSMIC and cfDNA datasets. **c** Distribution of primary tumor sites for significant mutations associated with these 36 genes

An integrated overview of somatic alterations and variants with significantly different frequencies between the AAM and CM cohorts is shown in Fig. 5a. The genes with the highest mutation frequencies across both groups were KMT2C, FANCA, KMT2D, PREX2, and ATM. Notably, the proportion of confirmed somatic mutations was higher than the proportion of differentially frequent variants identified in the preceding section, underscoring the robustness of these recurrent alterations. The distribution of primary tumor sites and associated histologies for the 36 genes is shown in Fig. 5b. Although these genes are not components of canonical prostate cancer signaling pathways, analysis of their primary site origins revealed that prostate tissue was the second most frequently represented tumor source. Notably, 22 of the 36 genes were identified in at least one sample originating from a prostate primary malignancy (Fig. 5c).

Although the shared SNPs identified between cfDNA and COSMIC are not directly linked to prostate cancer signaling pathways, their recurrent presence in prostate-derived samples highlights convergent mutational processes that may operate across multiple cancer types.

### Ancestry-informed characterization of cfDNA variations using gnomAD baselines reveals ethnicity-dependent mutational profiles

To contextualize the cfDNA variations detected in this study, we leveraged the gnomAD database, a widely used reference resource for evaluating population-specific allele frequency patterns. Allele frequencies from the Non-Finnish European (NFE) and African (AFR) cohorts were used as population baselines for CM and AAM patients, respectively, providing an ancestry-informed framework for comparison. This approach enabled a more refined assessment of race-associated differences within the mutational landscape. For each cfDNA mutation that demonstrated significant differential frequency between the two patient groups, the corresponding allele frequencies from the NFE and AFR populations were retrieved. The absolute difference between these values was quantified and designated as an ancestry informative marker (AIM). Variants with an AF difference greater than 0.01 were classified as exhibiting meaningful ancestry-related variability.

Five mutations exhibited markedly higher frequencies in AAM patients compared with CM patients. Notably, none of these variants had population-specific allele frequency data available in gnomAD (Table S1). Of these five mutations, two mapped to KMT2C, while the remaining three were associated with RB1, SETD2, and CHEK2P7.

To comprehensively assess ancestry-related mutational patterns between AAM and CM populations, we integrated three key parameters: (i) somatic status as defined by the COSMIC database, (ii) ancestry-related directionality based on alignment with population-specific allele frequencies, and (iii) involvement in the KEGG prostate cancer signaling pathway. Using these criteria, we categorized the SNPs into eight distinct groups for each population: Group 1) Aligned, somatic, and PC-related; Group 2) Aligned, somatic, and not PC-related; Group 3) Aligned, not somatic, and PC-related; Group 4) Aligned, not somatic, and not PC-related; Group 5) Not aligned, somatic, and PC-related; Group 6) Not aligned, somatic, and not PC-related; Group 7) Not aligned, not somatic, and PC-related; Group 8) Not aligned, not somatic, and not PC-related.

In this framework, Aligned designates SNPs in which the direction of differential frequency in cfDNA is concordant with the corresponding population-level allele frequency (AF), for example, a variant more common in AAM cfDNA is also more common in AFR relative to NFE by ≥ 0.01. Not Aligned indicates discordance between cfDNA dominance and population AF patterns. PC-related refers to SNPs located in genes annotated within the KEGG prostate cancer signaling pathway.

**Fig. 6:**
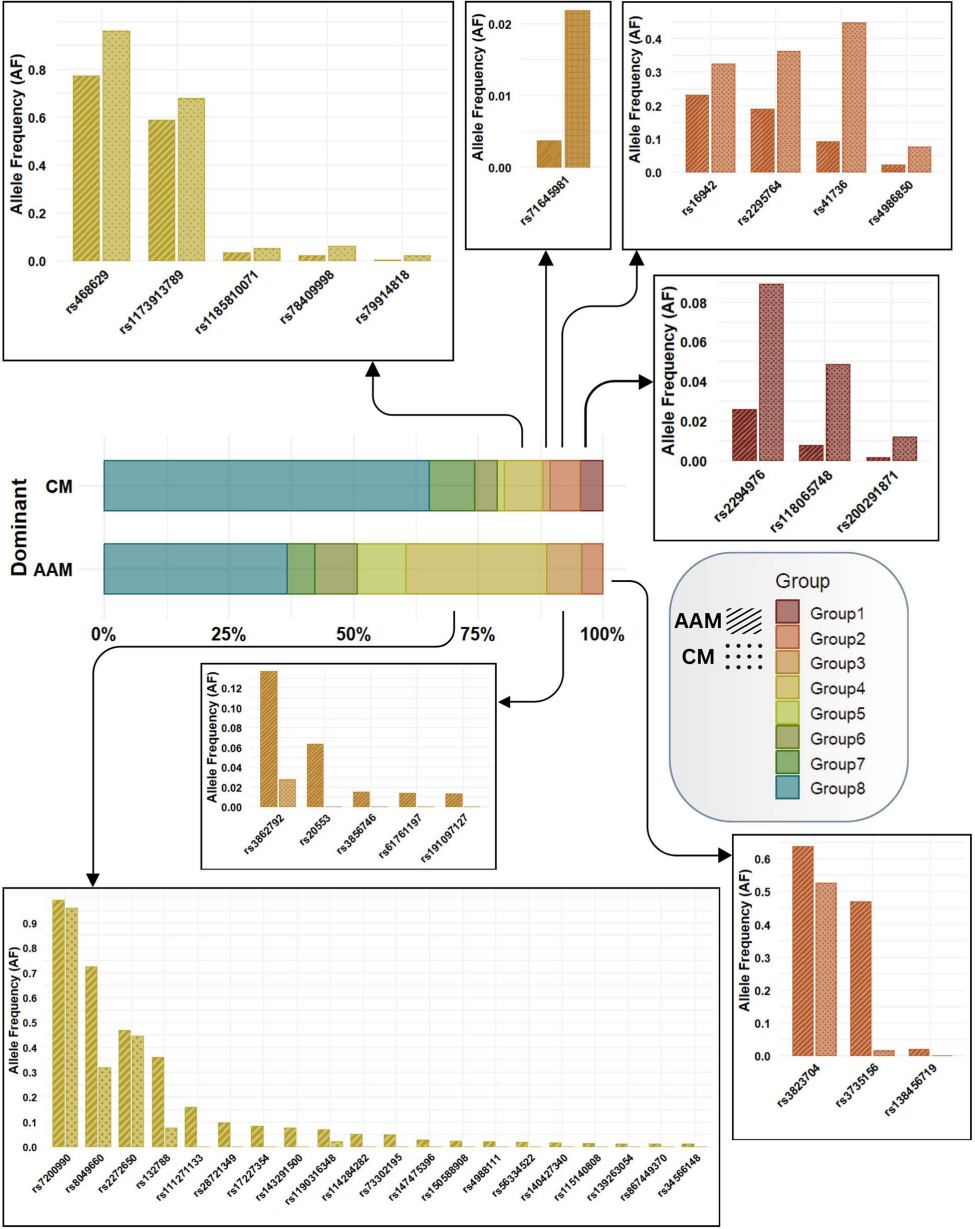
Classification of significant mutations into eight defined categories. Mutations were classified according to (i) the alignment of statistical significance with ancestry-related allele frequencies derived from the gnomAD database, (ii) somatic status, and (iii) their relevance to prostate cancer. The full description of the eight groups is provided in the main text

The distribution of SNPs across the eight resulting categories revealed marked population-specific patterns. Strikingly, Group 1 variants (aligned, somatic, and PC-related) were observed exclusively in CM patients (Fig. 6), suggesting that somatic alterations contributing to prostate cancer–related pathways may also be shaped by ancestry. Group 2 variants (aligned, somatic, but not PC-related) were detected in both populations, indicating shared somatic events that do not directly implicate canonical prostate cancer signaling. Group 4 variants (aligned, non-somatic, and not PC-related) were notably more frequent in AAM patients (*n* = 20) than in CM patients (*n* = 5), suggesting that ancestry-aligned germline variations unrelated to prostate cancer biology are more common in the AAM cohort. These variants likely reflect population-specific germline polymorphisms rather than somatic drivers or disease-associated alleles.

The remaining four categories (Groups 5–8), which encompass non-aligned variants, exhibited differing proportions across the two populations (Figs. S1 and S2), underscoring the complexity of ancestry-dependent mutational landscapes and their potential implications for interpreting cfDNA-based biomarkers.

### Comparative functional analysis of ethnic-specific mutations reveals divergent biological processes in prostate cancer

**Fig. 7:**
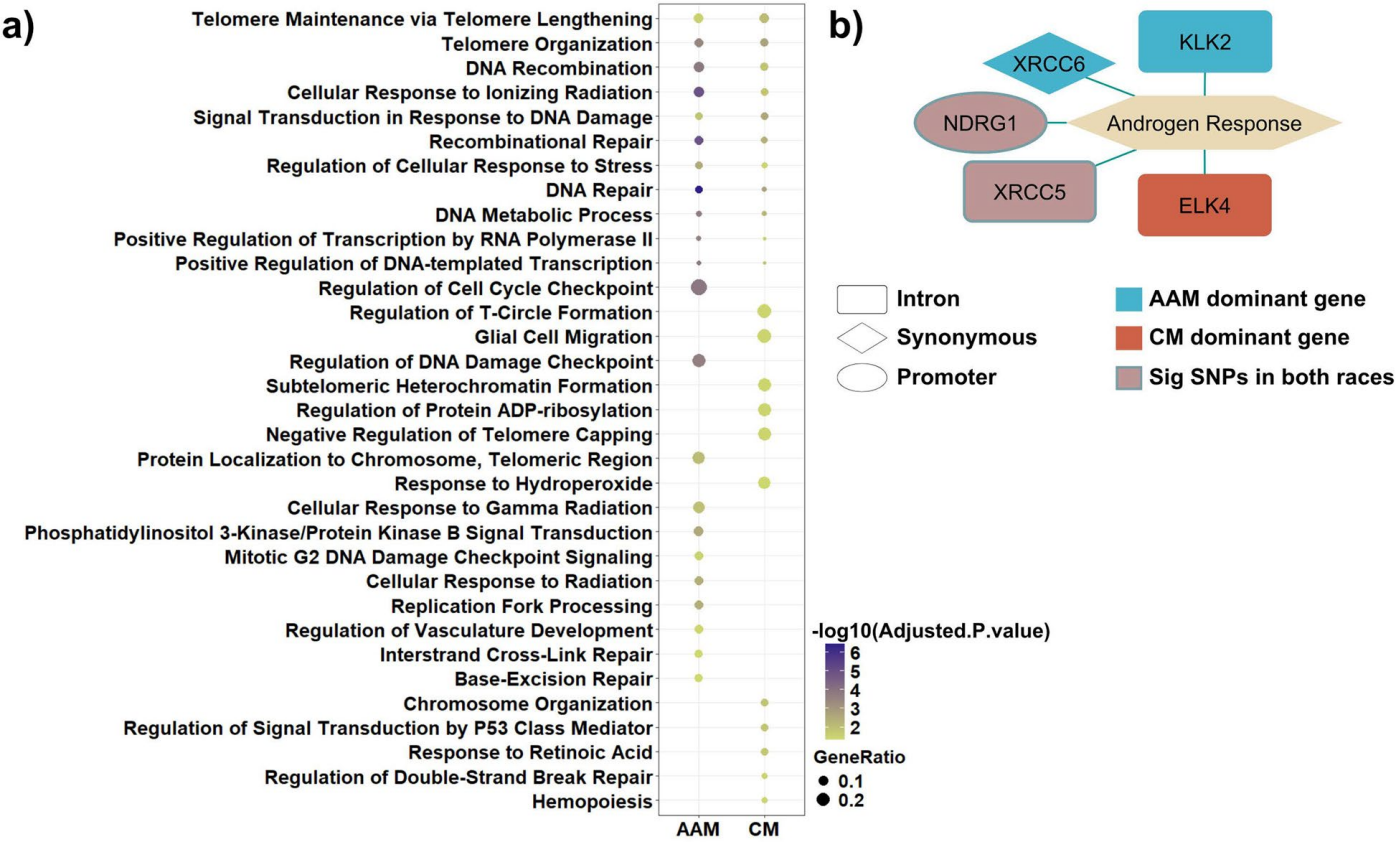
Functional enrichment of genes carrying race-specific significant mutations. **a** Enriched biological processes and **b** hallmark pathways derived from genes harboring significant variants that were not reported in COSMIC but exhibited differential frequencies between the two racial groups

In the final stage of the analysis, we examined mutations that were not represented in COSMIC yet were significantly differentially frequent between the two racial groups. This subset comprised 21 SNPs enriched in CM patients and 59 SNPs enriched in AAM patients. Genes associated with these variants were subjected to Gene Ontology (GO) enrichment analysis to assess biological process involvement, and pathway enrichment was performed using the MSigDB Hallmark collection. Eleven genes were shared across both groups ASXL1, ATM, CHEK2P2, DOT1L, ESRP1, KAT6A, KMT2CP4, NDRG1, RAD51, XRCC1, and XRCC5, indicating partial convergence despite overall divergence in mutational profiles. Notably, genes harboring ancestry-dominant mutations mapped to distinct biological processes, underscoring potential race-specific differences in the functional consequences of these alterations. CM-dominant mutations were enriched in biological processes related to chromosomal organization, telomere regulation, p53-mediated signaling, and oxidative or retinoic acid response pathways. By contrast, AAM-dominant mutations mapped predominantly to DNA damage response programs, cell-cycle checkpoint regulation, and PI3K–AKT signaling. Despite these divergent functional enrichments, both groups shared core genomic stability processes, including DNA repair, recombination, and telomere maintenance (Fig. 7a). Together, these findings reveal population-specific functional biases while highlighting a unifying reliance on DNA integrity–associated pathways across prostate cancer patients in both cohorts. Pathway enrichment analysis identified the androgen response as the only Hallmark pathway significantly associated with the input gene set (Fig. 7b). Within this pathway, XRCC6 and KLK2 exhibited predominant alterations in AAM patients, whereas ELK4 showed preferential variation in CM patients. XRCC5 and NDRG1 harbored mutations shared across both groups. These findings indicate that, although both populations demonstrated perturbations within the same androgen-responsive pathway, the specific genes affected, and therefore the molecular routes of pathway disruption, differed between racial groups.

## Discussion

In this study, we provided NGS profiling of cross-sectional plasma cfDNA samples obtained from mCRPC patients in two, AAM and CM racial groups. Although cfDNA genomic profiles reflect tumor heterogeneity, which is crucial for identifying resistant clones and selecting appropriate targeted therapies [11], we specifically focused on differences in genotyping between the two groups, particularly single nucleotide polymorphisms (SNPs). Our study expands the use of cfDNA profiling from detecting clinically actionable gene mutations to a genome-wide discovery tool for gaining deeper clinical and biological insights into advanced metastatic cancers. The cfDNA populations are typically complex and heterogeneous, as seen in the varied mutation patterns across different genes. Previous studies have shown that mutations in the *AKT* gene family and *mTOR* can enhance bone metastasis [12–15], aligning with our findings that the bone is a common site of metastasis in human prostate cancer [16]. Although we did not find significant differences in metastatic timing between the two racial groups.

This study offers an ancestry-informed, cfDNA-based delineation of the mutational landscape in mCRPC across AAM and CM patients. By integrating cfDNA-derived SNP profiles with curated reference datasets from COSMIC and gnomAD, we identified both shared and population-specific genomic alterations that may contribute to divergent disease trajectories. Although exploratory, these findings reveal notable ancestry-associated differences in the genetic architecture of metastatic prostate cancer and point toward the potential development of non-invasive, ancestry-tailored biomarkers to improve risk stratification and therapeutic decision-making.

The comparative analysis of cfDNA variants highlights significant ancestry-dependent differences in the molecular drivers perturbing key oncogenic signaling pathways [17], specifically the PI3K–AKT [18] and MAPK cascades. While both AAM and CM patients exhibit recurrent dysregulation of these pathways, the specific driver genes are distinct. AAM patients show dominant alterations in genes like AKT1, AKT3, MTOR, and AR [19], indicating molecular disruption primarily through these nodes. Conversely, CM patients preferentially harbor alterations in NKX3-1 [20], AKT2, and BRAF. This divergence suggests that despite similar pathways being affected (PI3K–AKT and MAPK), the precise "molecular nodes" of perturbation are population-specific. Since these cascades are critical for cell survival and resistance to therapy in prostate cancer, these findings underscore the need for ancestry-informed precision oncology. The differential patterns may reflect unique evolutionary or adaptive pressures, necessitating tailored diagnostic and treatment strategies for each population.

Our results highlight significant cross-pathway crosstalk between the PI3K–AKT survival signaling and p53/Cell Cycle regulatory networks in prostate cancer, suggesting that disease progression involves a complex balance between cell survival and genomic stability control [21, 22]. Within the Cell Cycle component, RB1 and CCNE exhibited somatic mutations. Notably, RB1 alterations were more prevalent in AAM patients. This finding is consistent with established literature linking RB1 loss to aggressive disease and poor prognosis in AAM prostate cancer [23]. The detection of RB1 somatic mutations in circulating cfDNA suggests its promising utility as a minimally invasive biomarker for assessing disease aggressiveness, particularly within the AAM population [23].

Our study highlights a differential representation of TMPRSS2, ERG, and ETV5 variants between AAM and CM. The well-established TMPRSS2–ERG fusion, found in ~ 40–50% of prostate cancer cases, promotes androgen-driven overexpression of ERG, driving transcriptional changes and tumorigenesis [24, 25]. Our cfDNA analysis showed TMPRSS2 variants were more prominent in AAM, whereas ETV5 variants were enriched in CM. The co-occurrence of somatic mutations in TMPRSS2 and ERG within cfDNA supports their functional relevance and suggests that distinct fusion-mediated transcriptional programs may operate across these populations. This finding is consistent with prior research indicating that ERG fusions are less frequent but more heterogeneous in AAM patients [26], potentially reflecting divergent mechanisms of androgen receptor pathway activation and emphasizing the need for population-specific therapeutic strategies.

A core set of five genes, KMT2C, FANCA, KMT2D, PREX2, and ATM, exhibited the highest shared mutation frequency between cfDNA and the COSMIC database across both AAM and CM cohorts. These genes are critically involved in chromatin remodeling, DNA repair, and genome stability, all fundamental processes for tumor evolution [27, 28]. The robust confirmation of these somatic mutations, supported by external data, suggests they possess significant pathogenic potential despite not being traditionally classified as canonical prostate cancer drivers. Their high COSMIC association with "prostate" as the second most frequent primary site, however, underscores a broader role in genomic instability and tumor maintenance.

Intriguingly, five specific mutations, including variants in KMT2C, RB1, SETD2, and CHEK2P7, were observed at significantly higher frequencies in AAM patients. A notable lack of corresponding allele frequency data in gnomAD reference populations suggests these may represent novel, potentially ancestry-specific mutations. Given the established tumor-suppressor function of RB1 and the epigenetic regulatory role of SETD2, their recurrent alteration in AAM cfDNA may highlight unique molecular vulnerabilities that could contribute to the known clinical disparities in this population. Future functional validation and replication in larger AAM cohorts are essential to determine their causal relevance and clinical utility as population-specific biomarkers.

Mutations that were aligned, somatic, and prostate cancer–related appeared predominantly in CM patients, suggesting that certain somatic alterations may be more strongly selected for, or more readily detectable, within European-derived genomic backgrounds. In contrast, aligned, non-somatic, and non–PC-related variants were markedly more frequent in AAM patients, indicating a greater prevalence of ancestry-aligned germline polymorphisms that are not directly oncogenic but may shape the underlying genomic landscape. This pattern is consistent with previous observations that African ancestry is associated with increased germline genetic diversity, which may in turn influence mutation burden, cfDNA composition, and variant detectability.

The non-aligned mutation groups exhibited varying proportions between the two populations, further emphasizing the complexity of ancestry-dependent mutational landscapes. Of particular interest were variants classified as somatic in COSMIC but more common in healthy NFE individuals within gnomAD, yet significantly enriched in AAM patients. These seemingly paradoxical patterns suggest that some variants may exert context-dependent functional effects influenced by underlying genomic ancestry. Such mutations could represent “ancestry-conditioned drivers,” whose oncogenic potential manifests only within specific genetic or epigenetic contexts.

Within the androgen response as the sole enriched hallmark pathway, XRCC6 and KLK2 exhibited predominant alterations in AAM patients, while ELK4 showed higher variation in CM patients. Interestingly, XRCC5 and NDRG1 were mutated in both groups, suggesting convergent pathway perturbation through distinct molecular routes. The androgen response pathway remains central to prostate cancer biology, and our findings indicate that ancestry-related differences may influence how androgen signaling dysregulation contributes to tumor progression. The identification of KLK2 and ELK4, genes with established roles in AR signaling and chromatin regulation [29, 30], as ancestry-biased candidates support their potential as targets for future mechanistic and translational research.

The integration of cfDNA profiling with population-specific allele frequency baselines allowed us to separate disease-associated somatic mutations from ancestry-linked germline variations. This distinction is particularly important in multi-ethnic biomarker studies, where failure to account for genetic background can lead to misclassification of benign polymorphisms as pathogenic alterations. Our analysis underscores that cfDNA contains a composite signal reflecting both tumor-derived mutations and inherited genomic features, and that ancestry-aware interpretation is essential for accurate biomarker discovery and clinical translation.

Although our sample size was modest and limited to a single institution, the consistency of ancestry-aligned mutational patterns across independent reference datasets provides confidence in the observed trends. Our study demonstrates the feasibility of leveraging cfDNA sequencing for comparative population genomics in cancer and highlights the necessity of integrating large-scale reference databases to control for confounding genetic diversity.

Several important limitations should be considered when interpreting our findings. First, the relatively small and imbalanced cohort (22 AAM vs. 66 CM) reduces statistical power and may limit the generalizability of ancestry-associated mutational patterns. Moreover, the cross-sectional nature of the cfDNA sampling precludes longitudinal assessment of mutation dynamics, clonal evolution, or treatment-related changes. Functional validation of the identified variants was also beyond the scope of this study; thus, the biological effects and mechanistic relevance of these alterations remain to be experimentally established. In addition, although COSMIC and gnomAD are widely used for variant interpretation, their representation of individuals with African ancestry remains incomplete, which may introduce bias into population-level comparisons and affect the accuracy of allele frequency–based classification.

Technical considerations further constrain the interpretation of our results. Although the Agilent SureSelect XT HS2 workflow incorporates unique molecular identifiers (UMIs) to reduce PCR-related artifacts, Partek Flow reports depth at the read level rather than at the level of unique molecular coverage (UMC). Given typical read-to-UMI ratios in cfDNA sequencing, the effective UMC is likely substantially lower than the nominal ~ 500 × read depth, resulting in an estimated limit of detection (LOD) of approximately 3–5% VAF. Many bona fide metastatic prostate cancer cfDNA mutations occur below this threshold; therefore, variants detectable in our dataset may be enriched for germline or clonal hematopoiesis (CHIP)–associated mutations. The absence of matched buffy-coat germline DNA further limits our ability to distinguish CHIP or germline variants from true tumor-derived events. Consequently, our findings should be interpreted as exploratory and hypothesis-generating. Future studies incorporating higher UMC depth, functional validation, and matched-normal sequencing will be essential to refine the biological and clinical significance of ancestry-associated cfDNA variation.

Future research should focus on expanding multi-ethnic cohorts, integrating transcriptomic and proteomic layers, and applying functional genomics approaches (e.g., CRISPR perturbation assays) to elucidate the mechanistic roles of ancestry-biased variants such as RB1, SETD2, and KMT2C. Additionally, prospective cfDNA monitoring in clinical trials could evaluate whether these variants correlate with treatment response or disease progression across diverse populations. Such studies will be critical for translating ancestry-informed genomics into equitable precision oncology.

In summary, this cfDNA-based analysis delineates distinct and shared genetic signatures of prostate cancer between African American and Caucasian patients. Our ancestry-aware framework, integrating COSMIC and gnomAD datasets, identified both known and novel variants that illuminate the molecular diversity of prostate cancer pathogenesis. The results underscore the need for population-informed approaches in cancer genomics and lay the groundwork for developing non-invasive, ancestry-tailored diagnostic and prognostic strategies.

## Methods

### Patient sample collection

We focused on AAM and CM patients because prostate cancer disproportionately affects these two groups, and they were the only populations in our cohort with adequate representation to enable robust, clinically interpretable comparisons. All patient samples used in this study were collected following the approval of our institution's ethical review board, the Indiana University Human Research Protection Program (HRPP), under protocol number 12515, and in strict compliance with ethical guidelines. Informed consent was obtained from all patients prior to sample collection, ensuring they were fully informed about the study's purpose, procedures, and potential risks. All samples were de-identified, and protected health information was managed in accordance with the Health Insurance Portability and Accountability Act (HIPAA) guidelines.

#### Inclusion criteria

Patients were eligible for inclusion if they: were adult male participants receiving care within the Indiana University clinical system; had a clinical diagnosis of prostate cancer documented in the medical record; self-identified race as AAM or CM/White (as recorded in the medical record and/or consent documentation); were able and willing to provide written informed consent; and were able to provide a peripheral blood specimen of sufficient volume for downstream analyses (10–20 mL). When available, relevant clinical annotations (e.g., age, PSA, Gleason/Grade Group, clinical stage, and treatment status) were recorded to support clinically interpretable comparisons.

#### Exclusion criteria

Patients were excluded if they: were unable or unwilling to provide informed consent; did not have a confirmed prostate cancer diagnosis; did not self-identify as AAM or CM/White (due to insufficient sample size for other groups in this cohort); had incomplete or missing key clinical annotation required for stratification (e.g., unknown disease status/stage when stratification was essential to the analysis); or had blood samples that were compromised or unsuitable for processing (e.g., insufficient volume, incorrect collection tube, gross hemolysis/clotting, delayed transport outside allowable handling conditions, or failure to meet pre-specified processing timelines/quality thresholds).

Peripheral blood (10–20 mL) was collected from patients into Streck Cell-Free DNA BCT® tubes. The tubes were gently inverted 8–10 times to ensure proper mixing of the anticoagulant. Samples were transported at room temperature and processed immediately. For plasma isolation, samples were centrifuged at 1600 × g for 10 min at 10 °C. The plasma supernatant was carefully transferred to new tubes and centrifuged again at 16,000 × g for 10 min to remove any remaining cellular debris. The plasma was aliquoted into sterile cryovials and stored at − 80 °C until further analysis.

### cfDNA purification

QIAamp MinElute ccfDNA Mini Kit (QIAGEN) was used to isolate cfDNA from patient samples. Up to 10 mL of plasma was collected and lysed with Proteinase K in the presence of magnetic beads, allowing cfDNA to bind efficiently. The beads, with bound DNA, were separated magnetically, and DNA was eluted into the buffer solution before further purification. The eluted DNA is then processed through QIAamp MinElute columns to adsorb on the silica membrane and remove contaminants. A final wash step was performed to eliminate residual contaminants, and DNA was eluted using ultra-clean water. The isolated DNA was in the 160–180 bp size range. Purified cfDNA was stored in −80 °C.

### Agilent SureSelect XTHS2 DNA NGS target enrichment

The quantity and quality of cfDNA samples were assessed using the Agilent TapeStation 4200, with DNA Integrity Numbers (DIN) ranging from 1.0 to 2.5 for the 91 samples analyzed. For library preparation, 10–20 ng of cfDNA was used. The process included end-repair, dA-tailing, adaptor ligation, and amplification, following the SureSelect XT HS2 DNA Library Preparation workflow (Version F0, May 2023, Agilent Technologies). PCR cycle numbers were adjusted according to the starting cfDNA amount: 10 cycles for 20 ng, 11 cycles for 10 ng, and 12 cycles for < 10 ng. The prepared libraries were hybridized, captured, and amplified using Agilent’s probe set (Product #5190–4812, 400 Kb, hg38). The final captured libraries were quantified and assessed for quality using Qubit and Agilent Bioanalyzer. Libraries were pooled in equimolar concentrations, with an average insert size of 250–300 bp. The pooled libraries were denatured, neutralized, and loaded onto the NextSeq 2000 platform for paired-end 150 bp sequencing (Illumina). Approximately 100 million reads were generated, achieving ~ 500-fold coverage. Over 90% of sequencing reads had a Phred quality score (Q score) of ≥ Q30, corresponding to 99.9% base call accuracy.

### Pipeline for sequencing quality control, alignment, variant calling, and annotation in cfDNA samples

The FASTQ files from plasma samples of PC patients were processed using Partek® Flow® software version 11.0.23.1204. The samples which had low sequencing quality or unknown racial information were excluded. We performed the alignment technique through the FASTQ files. In *Partek® Flow®* software, this task was executed via aligning reads to the hg38 reference genome using Burrows-Wheeler Alignment (BWA) [31] approach. We selected BWA-MEM which is faster and more accurate. The aligned reads were executed by several filter procedures: 1) duplicate filtering; 2) low mapping quality filtering while minimum mapping quality equaled 20; 3) failed platform/vendor quality reads filtering; 4) PCR/optical duplicates filtering.

The filtered reads were then subjected to variant calling using *FreeBayes * [32] v1.1.0 and *LoFreq * [33] v2.1.3 in *Partek® Flow®* software. All software versions mentioned are accurate as of the time of this study. Variants identified by both methods were retained for further analysis. The variants with depth lower than 199 or quality lower than 30 were excluded and the remaining ones were annotated using Ensemble Transcripts and dbSNP databases.

### Statistical methods for variant frequency comparison and mutation status analysis

To identify variants with significantly different frequencies between the two patient cohorts (AAM and CM), a Fisher's Exact Test was applied. This non-parametric test was chosen for its appropriateness in analyzing categorical data from small sample sizes and 2 × 2 contingency tables, where the cells represented the presence or absence of a specific variant in each of the two patient groups. The resulting *P*-values were adjusted to account for the issue of multiple comparisons inherent in large-scale variant analysis. The Benjamini-Hochberg (BH) procedure, which controls the False Discovery Rate (FDR), was utilized for this adjustment. Variants with an adjusted *P*-value (or *q*-value) of less than 0.05 (FDR < 0.05) were deemed to be statistically significant and carried forward for subsequent interpretation. Functional Consequence proportions were defined for all available variants, including those deemed significant in the comparison between patient groups.

### Curation of somatic mutation data from COSMIC

To establish a comprehensive catalogue of relevant somatic mutations, the Catalogue Of Somatic Mutations in Cancer (COSMIC) [34] v102 database was utilized as a curated and validated external reference. The initial dataset comprised 2,004,598 registered mutation data records derived from 1,558,299 samples across 7,125 distinct histological classifications. A rigorous filtering strategy was applied to tailor this expansive dataset specifically to the context of male prostate cancer and to generate a focused reference for downstream analysis.

### Exclusion criteria for prostate cancer context

The following sequential exclusion criteria were implemented to restrict the COSMIC data:

- Gender Restriction: Only mutation records derived from male samples were retained.
- Primary Site Exclusion: Mutation data associated with primary sites indicative of female reproductive cancers (e.g., ovary, breast, endometrium, cervix) were systematically excluded.
- Histology/Subtype Filtering: Primary histologies and specific subtypes unequivocally related to non-prostate cancers (e.g., sex cord-stromal tumor, ductal carcinoma, serous carcinoma) were filtered out.

The resulting subset of mutations, along with their associated metadata (including Primary Site, Histology, and specific mutation details), constituted the curated COSMIC reference database used for comparison with the study's cfDNA data.

### Rationale for broad tissue inclusion

Given the limited number of samples in the current study and the high proportion of metastatic disease at the time of cfDNA sampling, the precise organ-of-origin for all identified cfDNA mutations was uncertain. Therefore, the curated COSMIC reference included all remaining mutations observed across different tissues and cancers after the application of the above exclusionary filters. This approach was adopted to capture the full spectrum of potentially actionable and contextually relevant genomic instability drivers, regardless of the precise primary or metastatic site annotation within the COSMIC database.

### Reference population selection and allele frequency extraction

To identify potential ancestry-related variations among significant single nucleotide polymorphisms (SNPs) and establish population-specific allele frequencies (AFs), the Genome Aggregation Database (gnomAD) (v4.1) was utilized [35]. Due to the absence of a dedicated healthy control cohort in the present study, gnomAD served as the baseline reference for comparison.

The gnomAD African/African American (Afr) population subset was selected as the reference for the AAM patient cohort. Similarly, the Non-Finnish European (Nfe) population subset was used as the reference for CM patient cohort. AFs for the identified SNPs were extracted from the respective gnomAD populations.

### Rationale for gnomAD utilization

The gnomAD database, comprising sequence data from over 760,000 exomes and genomes, is widely accepted for its utility in genomic studies. The Nfe subset is a standard, broadly used healthy European reference population. Crucially, the gnomAD dataset is curated to exclude individuals with severe pediatric diseases, rendering it a suitable, large-scale control set for studying adult-onset conditions, such as prostate cancer. While gnomAD may contain some individuals with common adult diseases, its size and curation make it the most appropriate and robust public resource for assessing baseline population-specific AFs in the context of this study.

### Gene ontology and pathways enrichment analysis

Gene Ontology (GO) [36] enrichment analysis, specifically focusing on Biological Processes, was performed using the PANTHER [37] classification system. The background gene set for the analysis was established as Homo sapiens genes. Significance was determined using a hypergeometric test, and *P*-values were corrected for multiple testing using the Benjamini–Hochberg method. Pathway enrichment analysis was executed against the MSigDB Hallmark database. Additionally, given its manually curated and high-impact nature, the KEGG PATHWAY database was used for a separate, focused evaluation. This focused analysis specifically aimed to map the positions of all mutated genes, with a particular emphasis on mutations exhibiting racial-related frequency differences. Access to all mentioned enrichment databases (PANTHER, MSigDB, and KEGG) was facilitated through the Application Programming Interface (API) of web service via R packages, EnrichR, KEGGgraph, and KEGGREST. All network visualizations derived from the enrichment results were generated using Cytoscape software (version 3.10.4).

## Supplementary Information

Supplementary Material 1.

Supplementary Material 2.

Supplementary Material 3.

## Data Availability

All raw and processed sequencing data generated in this study are deposited to the SRA database under the accession number PRJNA1393153.
