# Supplementary material for: Circulating cell-free DNA profiling reveals ancestry-dependent genetic variation in metastatic prostate cancer
Source: Mol Biomed. 2026 Feb 12;7:13. doi: 10.1186/s43556-026-00405-8 (PMC12894460; doi:10.1186/s43556-026-00405-8)
Supplement: Supplementary file 1 — Supplementary Material 1. [file 43556_2026_405_MOESM1_ESM.docx]

**Supplementary Information for:**

**Circulating cell-free DNA Profiling Reveals Ancestry-Dependent Genetic Variation in Metastatic Prostate Cancer**

Samaneh Maleknia^1^, Rebecca Hassoun^1^, Nabil Adra^1,2^, Reza Shahbazi^1,2,3^*

^1^Division of Hematology/Oncology, Department of Medicine, Indiana University School of Medicine, Indianapolis, IN, USA

^2^Tumor Microenvironment & Metastasis, Indiana University Melvin and Bren Simon Comprehensive Cancer Center, Indianapolis, IN, USA

^3^Brown Center for Immunotherapy, Indiana University School of Medicine, Indianapolis, IN, USA

* Corresponding author: Reza Shahbazi

rshahbaz@iu.edu


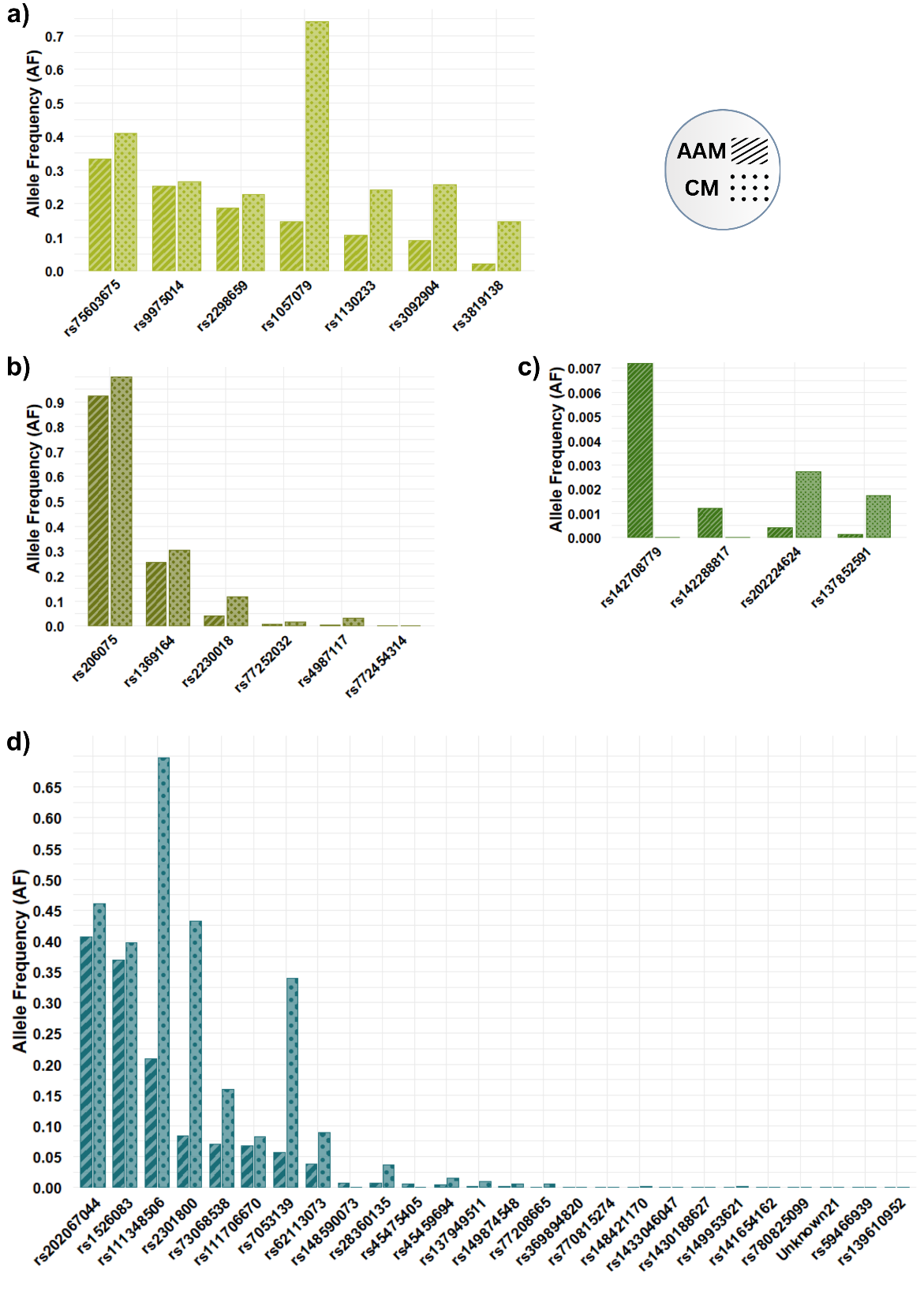


**Figure S1. Distribution of non-aligned cfDNA mutations in African American male (AAM) patients.** Bar plots illustrate the classification of cfDNA-derived mutations according to ancestry alignment, somatic status, and prostate cancer (PC) relevance: (a) Group 5 – non-aligned, somatic, and PC-related; (b) Group 6 – non-aligned, somatic, and not PC-related; (c) Group 7 – non-aligned, non-somatic, and PC-related; (d) Group 8 – non-aligned, non-somatic, and not PC-related.


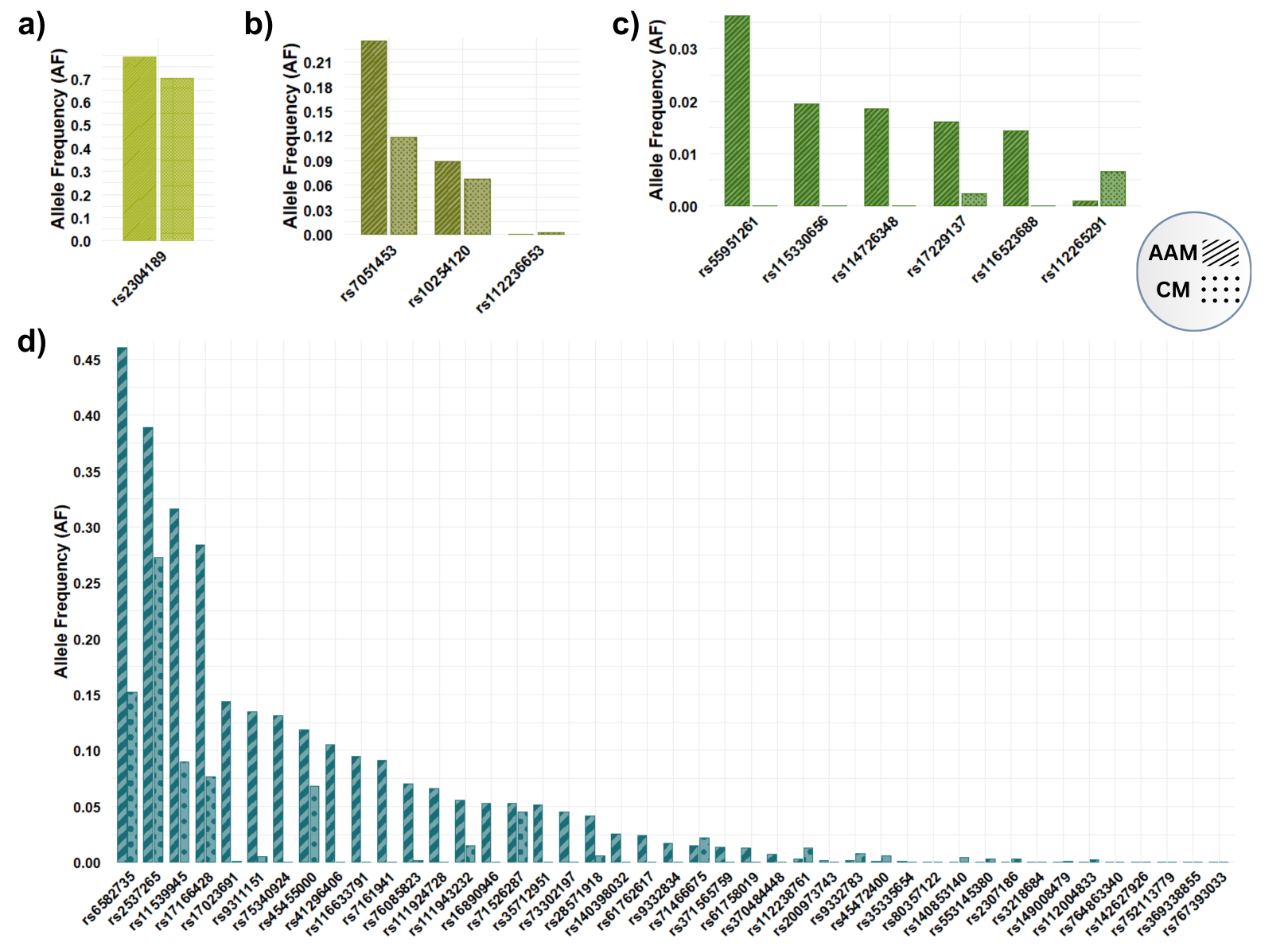


**Figure S2. Distribution of non-aligned cfDNA mutations in Caucasian male (CM) patients.** Bar plots depict the classification of cfDNA-derived mutations according to ancestry alignment, somatic status, and prostate cancer (PC) relevance: (a) Group 5 – non-aligned, somatic, and PC-related; (b) Group 6 – non-aligned, somatic, and not PC-related; (c) Group 7 – non-aligned, non-somatic, and PC-related; (d) Group 8 – non-aligned, non-somatic, and not PC-related.

**Table S1: Detailed information on all detected variants meeting the inclusion and exclusion criteria.** This table lists all identified mutations along with the number of total variants per gene and the number of mutations showing statistically significant differences between groups. For race-specific significant variants, allele frequency (AF) data were extracted from the gnomAD database, where Non-Finnish European (Nfe) and African/African American (Afr) allele frequencies were used as reference baselines for the Caucasian male (CM) and African American male (AAM) cohorts, respectively.

**Table S2. List of mutations shared between cfDNA and COSMIC datasets.** This table listed all mutations detected in cfDNA samples that were also identified in the COSMIC database, including primary site, sample and histology information.
